# An Autonomous Fruit and Vegetable Harvester with a Low-Cost Gripper Using a 3D Sensor

**DOI:** 10.3390/s20010093

**Published:** 2019-12-22

**Authors:** Tan Zhang, Zhenhai Huang, Weijie You, Jiatao Lin, Xiaolong Tang, Hui Huang

**Affiliations:** Guangdong Laboratory of Artificial Intelligence and Digital Economy (SZ), Shenzhen University, Shenzhen 510000, China; tan.zhang@utoronto.ca (T.Z.); zhenhaihuang0@gmail.com (Z.H.); youweijiee@gmail.com (W.Y.); zeup300@gmail.com (J.L.); tangtxl80@gmail.com (X.T.)

**Keywords:** harvesting robot, gripper, segmentation, cutting point detection

## Abstract

Reliable and robust systems to detect and harvest fruits and vegetables in unstructured environments are crucial for harvesting robots. In this paper, we propose an autonomous system that harvests most types of crops with peduncles. A geometric approach is first applied to obtain the cutting points of the peduncle based on the fruit bounding box, for which we have adapted the model of the state-of-the-art object detector named Mask Region-based Convolutional Neural Network (Mask R-CNN). We designed a novel gripper that simultaneously clamps and cuts the peduncles of crops without contacting the flesh. We have conducted experiments with a robotic manipulator to evaluate the effectiveness of the proposed harvesting system in being able to efficiently harvest most crops in real laboratory environments.

## 1. Introduction

Manual harvesting of fruits and vegetables is a laborious, slow, and time-consuming task in food production [1]. Automatic harvesting has many benefits over manual harvesting, e.g., managing the crops in a short period of time, reduced labor involvement, higher quality, and better control over environmental effects. These potential benefits have inspired the wide use of agricultural robots to harvest horticultural crops (the term crops is used indistinctly for fruits and vegetables throughout the paper, unless otherwise indicated) over the past two decades [2]. An autonomous harvesting robot usually has three subsystems: a vision system for detecting crops, an arm for motion delivery, and an end-effector for detaching the crop from its plant without damaging the crop.

However, recent surveys of horticultural crop robots [3,4] have shown that the performance of automated harvesting has not improved significantly despite the tremendous advances in sensor and machine intelligence. First, cutting at the peduncle leads to higher success rates for crop detachment, and detachment at the peduncle reduces the risk of damaging the flesh or other stems of the plant and maximizes the storage life. However, peduncle detection is still a challenging step due to varying lighting conditions and occlusion of leaves or other crops [5], as well as similar colors of peduncle and leaves [6,7]. Second, existing manipulation tools that realize both grasping and cutting functions usually require a method of additional detachment [8,9], which increases the cost of the entire system.

In this paper, we present a well-generalized harvesting system that is invariant and robust to different crops in an unstructured environment aimed at addressing these challenges. The major contributions of this paper are the following: (i) development of a low-cost gripper (Supplementary Materials) that simultaneously clamps and cuts peduncles in which the clamper and cutter share the same power-source drive, thus maximizing the storage life of crops and reducing the complexity and cost; (ii) development of a geometric method to detect the cutting point of the crop peduncle based on the Mask Region-based Convolutional Neural Network (Mask R-CNN) algorithm [10].

The rest of this paper is organized as follows: In Section 2, we introduce related work. In Section 3, we describe the design of the gripper and provide a mechanical analysis. In Section 4, we present the approach of cutting-point detection. In Section 5, we describe an experimental robotic system to demonstrate the harvesting process for artificial and real fruits and vegetables. Conclusions and future work are given in Section 6.

## 2. Related Work

During the last few decades, many systems have been developed for the autonomous harvesting of soft crops, ranging from cucumber [11] and tomato-harvesting robots [12] to sweet-pepper [2,13] and strawberry-picking apparatus [14,15]. The work in [16] demonstrated a sweet-pepper-harvesting robot that achieved a success rate of 6% in unstructured environments. This work highlighted the difficulty and complexity of the harvesting problem. The key research challenges include manipulation tools along with the harvesting process, and perception of target fruits and vegetables with the cutting points.

### 2.1. Harvesting Tools

To date, researchers have developed several types of end-effectors for autonomous harvesting, such as suction devices [17], swallow devices [18], and scissor-like cutters [15]. The key component of an autonomous harvester is the end-effector that grasps and cuts the crop. A common approach for harvesting robots is to use a suction gripping mechanism [16,19,20] that picks up fruit by pushing the gripper onto the crop to generate air to draw a piece of crop into the harvesting end and through the tubular body to the discharge end. These types of grippers do not touch the crops during the cutting process, but physical contact occurs when they are inhaled and slipped into the container, potentially causing damage to the crops, especially to fragile crops. Additionally, the use of additional pump equipment increases the complexity and weight of the robotic system, resulting in difficulties for compact and high-power density required in autonomous mobile robots [21,22]. Some soft plants, such as cucumbers and sweet peppers, must be cut from the plant and thus require an additional detachment mechanism and corresponding actuation system. The scissor-like grippers [16,23] that grasp the peduncles or crops and cut peduncles can be used for such soft plants. However, these grippers are usually designed at the expense of size. A custom harvesting tool in [16] can simultaneously envelop a sweet pepper and cuts the peduncle with a hinged jaw mechanism; however, size constraints restrict its use for crops with irregular shapes [24].

To overcome these disadvantages, we have designed a novel gripper that simultaneously clamps and cuts the peduncles of crops. In addition, the clamping and detachment mechanism share the same power-source drive rather than using separate drives. The benefits of sharing the same power source for several independent functions are detailed in our previous work [25,26]. Since the gripper clamps the peduncle, it can be used for most fruits that are harvested by cutting the peduncle. Since the gripper does not touch the flesh of the crop, it can maximize the storage life and market value of the crops. As the clamping and cutting occurs simultaneously at the peduncle, the design of the gripper is relatively simple and therefore cost-effective.

### 2.2. Perception

Detection in automated harvesting determines the location of each crop and selects appropriate cutting points [1,27,28] using RGB cameras [29,30] or 3D sensors [13,15]. The 3D localization allows a cutting point and a grasp to be determined. Currently, some work has been done to detect the peduncle based on the color information using RGB cameras [6,7,29,30]; however, such cameras are not capable of discriminating between peduncles, leaves, and crops if they are in the same color [7]. The work in [31] proposed a dynamic thresholding to detect apples in viable lighting conditions, and they further used a dynamic adaptive thresholding algorithm [32] for fruit detection using a small set of training images. The work in [27] facilitates detection of peduncles of sweet peppers using multi-spectral imagery; however, the accuracy is too low to be of practical use. Recent work in deep neural networks has led to the development of a state-of-the-art object detector, such as Faster Region-based CNN (Faster R-CNN) [33] and Mask Region-based CNN (Mask R-CNN) [10]. The work in [34] uses a Faster R-CNN model to detect different fruits, and it achieves better performance with many fruit images; however, the detection of peduncles and cutting points has not been addressed in this work. To address these shortcomings, we use a Mask R-CNN model and a geometric feature to detect the crops and cutting points of the peduncle.

## 3. System Overview and the Gripper Mechanism

### 3.1. The Harvesting Robot

**Figure 1:**
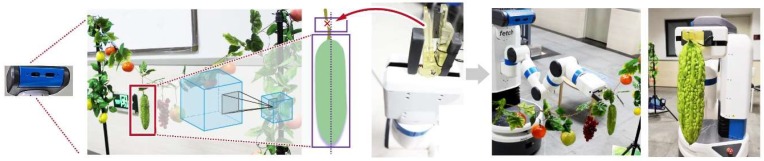
Architecture of the proposed harvesting robot.

The proposed harvesting robot has two main modules: a detection module for detecting an appropriate cutting point on the peduncle, and a picking module for manipulating crops (see Figure 1). Specifically, the detection module determines the appropriate cutting points on the peduncle of each crop. The picking module controls the robot to reach the cutting point, clamps the peduncle, and cuts the peduncle. The clamping and cutting actions are realized by the newly designed gripper.

### 3.2. The Gripper Mechanism

**Figure 2:**
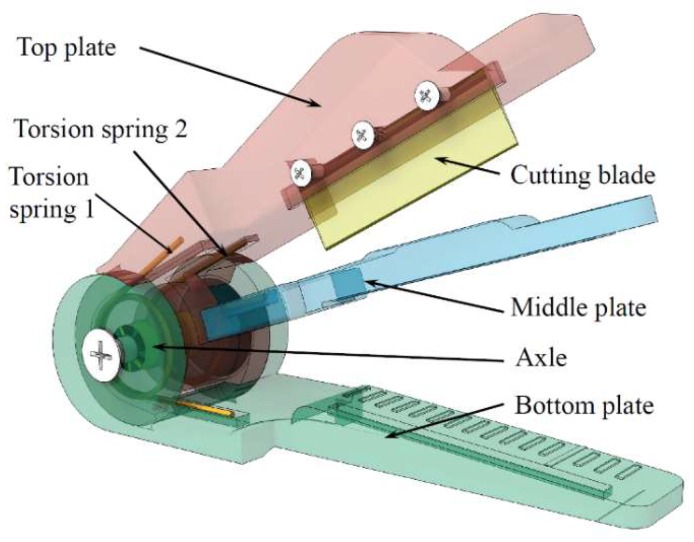
The 3D view of the proposed gripper [35,36].

The proposed gripper is illustrated in Figure 2 from a 3D point of view. Inspired by skilled workers who use fingers to gently hold the peduncles and use their fingernails to cut the peduncles, this gripper is designed with both clamping and cutting functions with three key parts only: a top plate with a cutting blade, a middle plate to press the peduncle, and a bottom plate to hold the peduncle. Two torsion springs and an axle are included.

**Figure 3:**
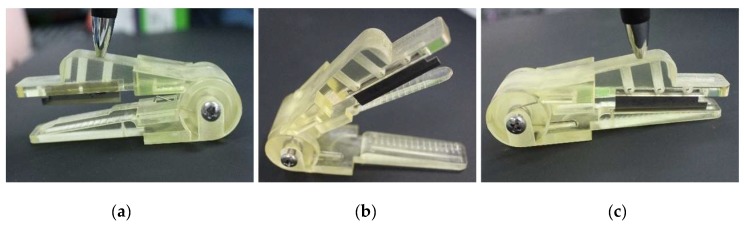
Cutting process: (**a**) rest position; (**b**) clamping state; (**c**) cutting state.

The clamping and cutting processes work as follows:

1. Clamping the peduncle: pressing the top plate, the middle plate contacts the bottom plate based on “*torsion spring 2*”, and thus the peduncle is clamped, as shown in Figure 3b.
2. Cutting the peduncle: continuing to press the top plate, the cutting blade contacts the peduncle on the bottom plate, thereby cutting the peduncle, as shown in Figure 3c.

After releasing the top plate, the top and middle plates are automatically restored to the rest position (Figure 3a) by “*torsion springs 1 and 2*”, respectively.

### 3.3. Force Analysis of the Gripper

**Figure 4:**
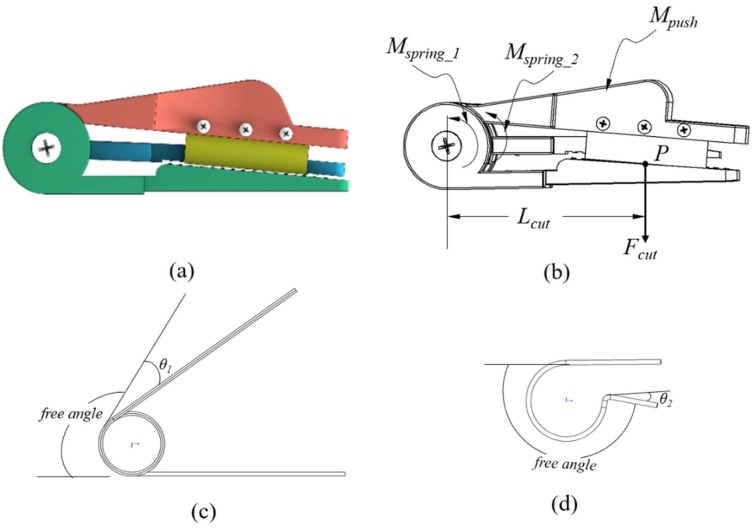
Force diagram of the cutting blade touching the bottom plate. (**a**) The diagram of the gripper. The force analysis of the gripper (**b**), *Torsion spring* 1 (**c**), and *Torsion spring* 2 (**d**).

As shown in Figure 4, when the cutting blade touches the bottom plate, the cutting force on the peduncle is calculated using the following equations:

(1) $$M_{cut}=\;M_{push}-M_{spring\_1}-M_{spring\_2}$$

(2) $$M_{spring\_1}\;=\;k_{1\;}\cdot \theta_{1}\;$$

(3) $$M_{spring\_2}\;=\;k_{2\;}\cdot \theta_{2}$$

where *M_cut_*, *M_push_*, *M_spring_1_*, *M_spring_2_* are the moments of cutting force, pushing force, spring 1 and spring 2. *k*_1_ and *k*_2_ are the torsion coefficients of “torsion springs 1 and 2”, respectively. *θ*_1_ and *θ*_2_ are the angles of deflection from the equilibrium position of “torsion springs 1 and 2”, respectively.

“*Torsion spring 1*” is the spring between the top plate and bottom plate, while “*torsion spring 2*” is the spring between the top plate and middle plate; see also Figure 2 and Figure 4d. With the moment *M_cut_*, the cutting force *F_cut_* can be described as:

(4) $$M_{cut}\;=\;F_{cut}\cdot L_{cut}$$

where *F_cut_* is the force applied on the peduncle from the cutting blade, *L_cut_* is the length between the axis of the coil and the line of the cutting force, see Figure 4b. *P* is the contacting point between the cutting blade and the peduncle.

Thus, $F_{cut}$ is derived from Equations (1)–(4) as follows:

(5) $$F_{cut}\;=\;\;\frac{M_{push}-k_{1\;}\cdot \theta_{1}-k_{2\;}\cdot \theta_{2}}{L_{cut}}$$

For further investigating the cutting force on peduncle, we analyzed the stress:

(6) $$F_{cut}\;=\;\tau_{cut}\cdot A$$

(7) $$\tau_{cut}\;=\;\frac{M_{push}-k_{1\;}\cdot \theta_{1}-k_{2\;}\cdot \theta_{2}}{A\cdot L_{cut}}$$

where *τ_cut_* is the stress, and *A* is the surface area of the peduncle. The two variables determine if the peduncle can be cut off. Equation (7) is derived from Equations (5) and (6).

**Figure 5:**
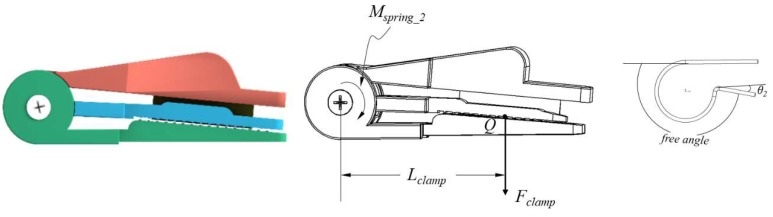
Force diagram of the middle plate touching the bottom plate.

As shown in Figure 5, when pressing the top plate and the middle plate touches the bottom plate, “*torsion spring 2*” creates the pressing force on the middle plate, and thus generates force on the bottom plate. This force ensures that the gripper clamps the peduncle before the cutting blade touches the peduncle:

(8) $$M_{clamp}\;=\;M_{spring\_2}$$

(9) $$M_{clamp}\;=F_{clamp}\cdot L_{2}$$

(10) $$M_{spring\_2}=k_{2\;}\cdot \theta_{2}$$

After the peduncle is cut, the crop is detached from the peduncle if the clamping force is smaller than the gravity of the crop. To ensure that the crop is clamped, the minimum clamping force overcoming the gravity is expressed as:

(11) $$F_{clamp}\geq \frac{m\cdot g}{\mu }$$

where *m* is the weight of the crop, *g* the gravity, and *µ* the coefficient of friction for a crop on the gripper plate surface. Based on the Equations (8)–(11), we obtain the minimum coefficient of “*torsion spring 2*”:

(12) $$k_{2\;}\geq \frac{m\cdot g\cdot L_{clamp}}{\mu \cdot \theta_{2}\;\;}$$

## 4. Perception and Control

### 4.1. Cutting-Point Detection

**Figure 6:**
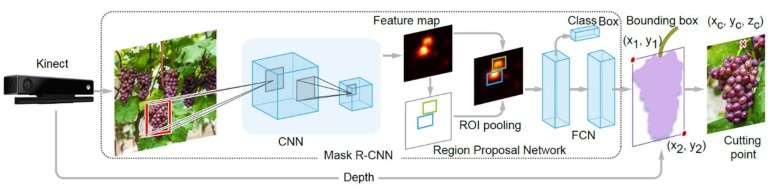
Illustration of the vision system that detects and localizes the crop and its cutting point.

The approach to detecting the cutting points of the peduncles consists of two steps: (i) the pixel area of crops is obtained using deep convolutional neural networks; (ii) the edge images of fruits are extracted, and then a geometric model is established to obtain the cutting points. Here, we obtain the external rectangle size of each pixel area to determine the region containing the peduncle for each crop. The architecture of the cutting-point-detection approach is shown in Figure 6.

Accurate object detection requires detecting not only which objects are in a scene, but also where they are located. Accurate region proposal algorithms thus play significant roles in the object-detection task. Mask R-CNN [10] makes use of edge information to generate region proposals by using the Region Proposal Network (RPN). Mask R-CNN uses color images to perform general object detection in two stages: region proposal, which proposes candidate object bounding boxes, and a region classifier that predicts the class and box offset with a binary mask for each region of interest (ROI). To train Mask R-CNN for our task, we perform fine-tuning [37], which requires labeled bounding-box information for each of the classes to be trained. After extracting the bounding box for each fruit, the geometric information of each region (e.g., external rectangle) is extracted by geometric morphological calculation. The cutting region of the interest of the targeted crop can thus be determined. A RGB-D camera like the Kinect provides RGB images along with per-pixel depth information in real time. With the depth sensor, the 2D position of the cutting point is mapped to the depth map, and thus the 3D information of the cutting point (x,y,z) is obtained.

**Figure 7:**
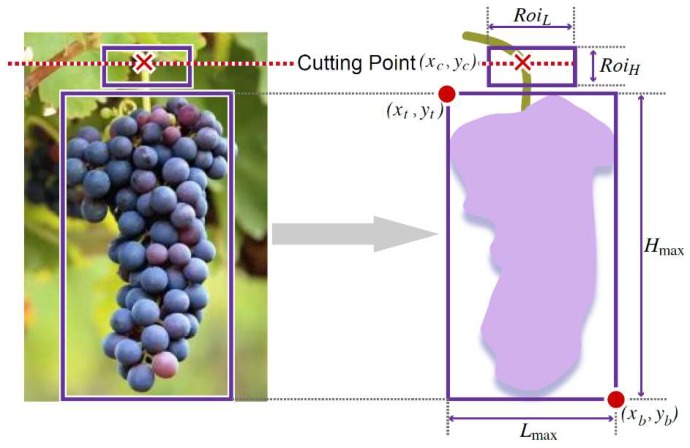
A schematic diagram of the cutting-point-detection approach.

The peduncle is usually located above the fruit. Thus, we set the ROI of the peduncle above the fruit, as shown in Figure 7.

The directions of the crops can be downward or tilted. We also adapted the minimum bounding rectangle to Mask R-CNN, thus to obtain the bounding boxes for crops in tilted direction. Other cases such as the direction of the crop parallel to the camera or the peduncle being “occluded” by its flesh will be discussed in the future work.

We then obtain the bounding box of the fruit, i.e., a top point coordinate (*x_t_, y_t_*), and a bottom point coordinate (*x_b_, y_b_*). The coordinates of the cutting point (*x_c_, y_c_*) can be determined as follows:

*Roi_L_* = 0.5·*L_max_* (13)

*Roi_H_* = 0.5·*H_max_* (14)

*L_max_* = |*x_b_ − x_t_*| (15)

*H_max_* = |*y_b_ − y_t_*| (16)

*x_c_ = 0.5(x_b_ − x_t_) ± 0.5Roi_L_* (17)

*y_c_ = y_t_ − 0.5Roi_H_* (18)

### 4.2. System Control

A common method of motion planning for autonomous crop harvesting is open-loop planning [16,38]. Existing work on manipulation planning usually requires some combination of precise models, including manipulator kinematics, dynamics, interaction forces, and joint positions [39]. The reliance on manipulator models makes transferring solutions to a new robot configuration challenging and time-consuming. This is particularly true when a model is difficult to define, as in custom harvesting robots. In automated harvesting, a manipulator is usually customized to a special crop; thus, they are not as accurate as modern manipulators and it is relatively difficult to obtain accurate models of the robots. In addition, requiring high position sensing becomes challenging in manipulators given the space constraints.

In this paper, we demonstrate a learning-based kinematic control policy that maps target positions to joint actions directly, using convolutionally neural network (CNN) [40]. This approach makes no assumptions regarding the robot’s configuration, kinematic, and dynamic model, and has no dependency on absolute position sensing available on-board. The inverse-kinematics function is learned using CNNs, which also considers self-collision avoidance, and errors caused by manufacturing and/or assembly tolerances.

**Figure 8:**
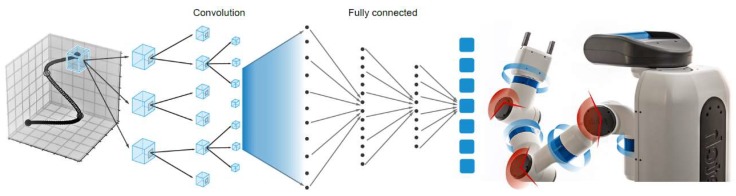
Network architecture for the kinematic control of a robotic manipulator.

As shown in Figure 8, the network comprises three convolutional layers and three fully connected layers. The input is the spatial position of the end-effector, and the output is the displacement of all of the joints. The fully connected layers will serve as a classifier on top of the features extracted from the convolutional layers and they will assign a probability for the joint motion being what the algorithm predicts it is.

We collected data by sending a random configuration request ***q*** = (*q*_1_, *q*_2_, …, *q*_*n*_) to a robot with *n* joints and observing the resulting end-effector position ***x*** = (*x*_1_, *x*_2_, …, *x*_*n*_) in 3D space. *n* is the number of joints. This results in a kinematic database with a certain number of trails:

*X* = (< ***q***_1_, ***x***_1_>, < ***q***_2_, ***x***_2_ >, …, < ***q***_n_, ***x***_n_ >) (19)

We use the neural network shown in Figure 8 to estimate the inverse kinematics from the collected data. Then we generate a valid configuration ***q***_*n*_ for a given target position ***x***_*n*_. ***q***_*n*_ and ***x***_*n*_ are the *n_th_* set of training data. We use this approach for planning to reach a cutting point of the peduncle.

## 5. Hardware Implementation

**Figure 9:**
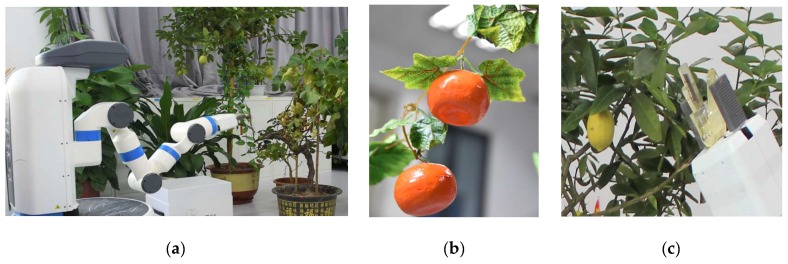
Setup for the experiments. (**a**) The experiment setup; (**b**) The plastic crops with plastic peduncles hung on a rope; (**c**) The proposed gripper mounted on the end-effector of the Fetch robot.

The following experiments aim at evaluating the general performance of the harvesting system. As shown in Figure 9, the proposed gripper was mounted on the end-effector of the Fetch robot (see Figure 9c). The Fetch robot consists of seven rotational joints, a gripper, a mobile base, and a depth camera. The depth camera was used to detect and localize the crops. The robot is based on the open source robot operating system, ROS [41]. The transformation between the image coordinate frame and the robot coordinate frame are obtained with ROS packages. The three plates and the shaft of the proposed gripper are created using Polylactic Acid (PLA) with a MakerBot Replicator 2 printing machine. The length, width, and height of modules are taken as 11, 5, and 9 cm, respectively.

Since the Fetch robot is not suitable for working in the field, the experiments were conducted in a laboratory environment. In order to verify the effectiveness of the proposed system in identifying and harvesting a variety of crops, we use both real and plastic ones, as shown in Figure 9a,b. Two experiments are presented in the following section aimed at validating the perception system and the harvesting system. First, we present the accuracy of the detection of crops and the cutting points. Second, we present the experiment of the full harvesting platform, demonstrating the harvesting performance of the final integrated system.

### 5.1. Detection Results

Seven types of crops were tested, including lemons, grapes, common figs, bitter melon, eggplants, apples, and oranges. For each type of crop, we used 60 images for training and noises were introduced in the images. The number of images was inspired by Sa et al. [34]. We collected images from Google and set labelled bounding box information for each crop. The images taken with the Kinect sensor were used for testing only. In this paper, we used 15 testing images for each type of crop. These images are taken from different viewpoints. We have one plant for each type of the real fruits.

**Figure 10:**
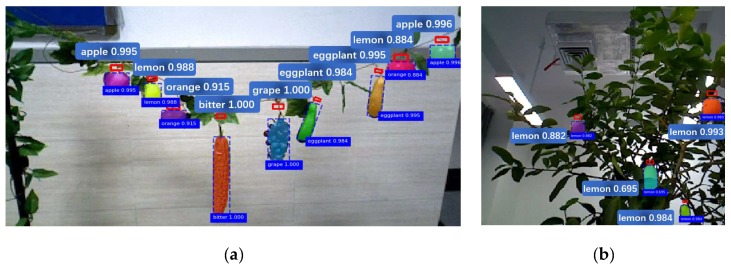
Results of the cutting-point-detection for some plastic (**a**) and real crops (**b**).

An example is shown in Figure 10 where the real crops were on the plants (see Figure 9a). For a convenient harvesting, all the plastic crops were hung on a rope, as shown in Figure 9b. We had three attempts and the crops were repositioned the crops every time. Therefore, we used 27 plastic crops (six apples, three lemons, six oranges, three bitter melons, three clusters of grapes, and six eggplants) and 31 real crops (seven grape bunches, 14 lemons, and 10 common figs).

**Figure 11:**
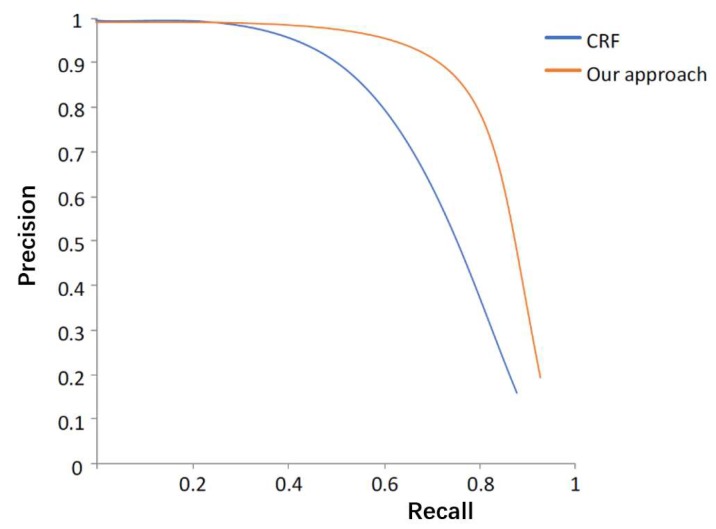
Precision-recall curve for detecting lemons using the CRF-based approach and the proposed approach.

We utilize the precision-curve [42] as the evaluation metric for the detection of cutting points of lemons. The performance of the proposed detection method and the baseline method Conditional Random Field (CRF) [43] was compared. Figure 11 shows that the two methods have similar performance. It can be seen that there is a gradual linear decrease in precision with a large increase in recall for the proposed approach.

**Figure 12:**
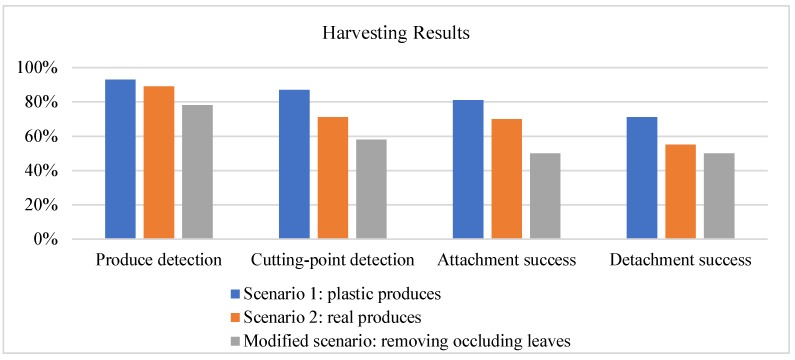
Harvesting rates for autonomous harvesting experiment.

Some of the detection results for real and plastic crops were shown in Figure 10. The results show that the detection success rates for plastic and real crops are 93% (25/27) and 87% (27/31), respectively. The detection success rates for the cutting points for plastic and real crops are 89% (24/27) and 71% (22/31), respectively. The result can be seen in Figure 12.

### 5.2. Autonomous Harvesting

Motion planning is performed for autonomous attachment and detachment. The attachment trajectory starts at a fixed offset from the target fruit or vegetable determined by the bounding box. Then, the trajectory makes a linear movement towards the cutting point. The whole motion was computed using the control policy that maps target positions to joint actions introduced in Section 4.2. Self-collisions of the robot were included within the motion planning framework to ensure the robot safely planned. Once the attachment trajectory has been executed attaching the peduncle, the end-effector is moved horizontally to the robot from the cutting point.

A successful harvest consists of a successful detection of the cutting point and a successful clamping and cutting of the peduncle. The crop that was successfully picked was replaced with a new one hung on the rope. We repositioned the crop for repicking if it was not harvested successfully. For real crops, the robot harvests each crop once, including seven grape bunches, 14 lemons, and 10 common figs. Similar to plastic fruits, reaching the crop’s cutting point is counted as a successful attachment, and clamping and cutting of the peduncle is counted as a successful detachment. Results show that most plastic fruits and vegetables were successfully harvested. The result is shown in Figure 12. The detailed success and failure cases can be seen in Table 1. The attachment success, i.e., the success rate of the robot’s end-effector reaching the cutting point is 81% and 61% for plastic and real crops, respectively. The detachment success rates are 67% and 52%, respectively.

**Figure 13:**
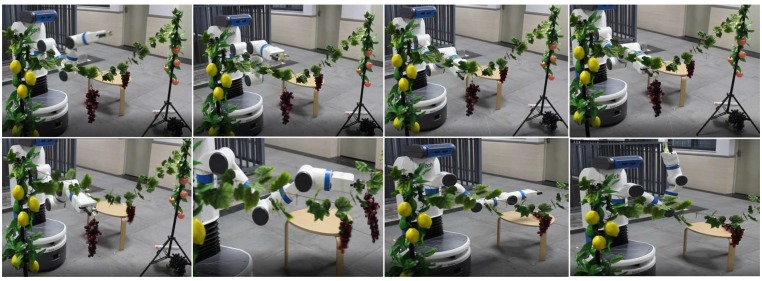
Video frames showing the robot reaching a cluster of plastic grapes, cutting the peduncle, and placing them onto a table.

**Figure 14:**
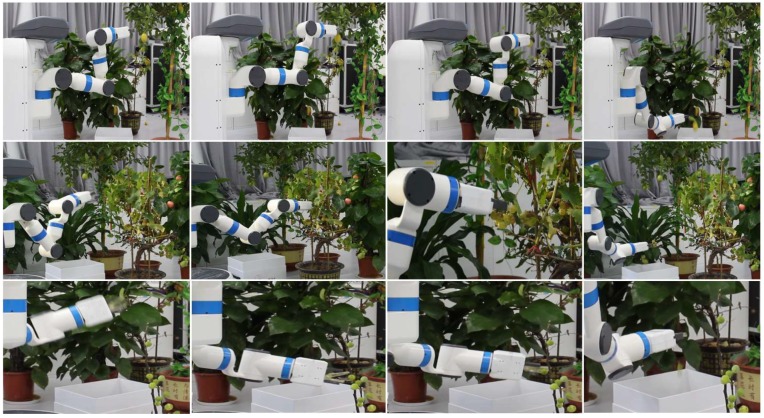
Video frames showing robot harvests real lemons (top), grapes (middle), and common figs (bottom).

Figure 13 is the video frames showing the robot reaching a cluster of plastic, cutting the peduncle, and placing them onto the desired place. Figure 14 is the video frames showing the robot harvesting real lemons, grapes and common figs in the laboratory environment. A video of the robotic harvester demonstrating the final experiment by performing autonomous harvesting of all crops is available at http://youtu.be/TLmMTR3Nbsw.

**Figure 15:**
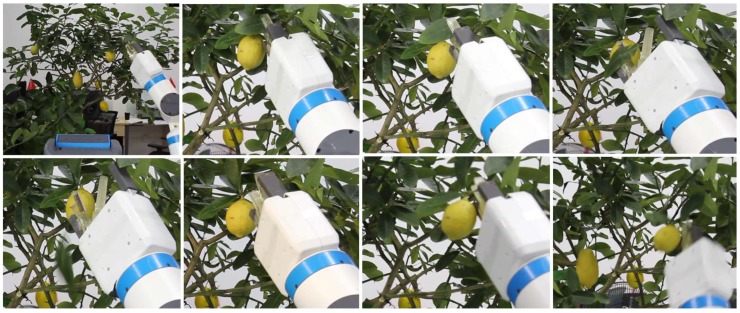
Video frames for the modified scenario in which the robot picked an occluded lemon (bottom) by removing an occluding leaf (top).

For cases where the crop’s cutting point is occluded by leaves, the robot can remove the leaves to make the crop’s cutting point visible. First, the overlap between crop’s cutting area and the leaf is calculated to see if it exceeds a given threshold. Then, the robot detects the leaf’s cutting point and cuts it using the same method as that for cutting crops. Figure 15 shows a modified scenario in which the robot removed leaves that occluded the fruits. We made four attempts and failed twice. The figure shows that the leaf stuck onto the cutting blade and it did not detach even if the gripper is open.

### 5.3. Discussion

A failure analysis was conducted to understand what were the major causes of harvesting failures within the system proposed, and thus to improve the system in the future work. The mode of failures includes: (1) fruits or vegetables not detected or detected inaccurately due to occlusions; (2) cutting point not detected due to the irregular shapes of the crops; (3) peduncle not attached due to motion planning failure; (4) peduncle partially cut since the cutting tool stuck into the peduncle; (5) crop dropped since the gripper does not clamp the crop tightly; (6) detachment fails since the crops or leaves stays on the blade when the gripper is open.

Figure 11 shows the success rates for three scenarios: Scenario 1 with plastic crops, Scenario 2 with real crops, and a modified scenario in which the robot picked crops by removing occlusions. The detection of the fruits and the cutting-points, attachment, and detachment harvest success rate were included. The detachment success rate reflects the overall harvesting performance.

The results showed that Scenario 1 with plastic crops has a higher harvest success. The most frequent failure mode was (2) cutting point not detected and (5), occurring 30% harvest failure. For instance, the two attachment failures (orange 1 and eggplant 1) occurred in the first nine crops. For orange 1, the cutting blade missed the peduncle as the peduncle is short and the cutting blade missed it. Eggplant 1 was irregularly shaped, which resulted in a poor cutting-pose estimation. We then adjusted the directions of the orange and eggplant for the following two rounds, and there was no detection error. The lower detachment success rate is most likely attributed to the grapes and bitter melon since they have a larger weight and fall.

The harvest success rate (55%) was lower for Scenario 2 with real crops. The most frequent failure modes were (4), and (5) which represented a significant portion of the failure cases and showed that attachment and detachment process is challenging due to the lower stiffness of the cutting mechanism, as well as the sophisticated environment. For instance, the common figs have shorter peduncles and the gripper is thick and wider, resulting in a detachment failure. The grape peduncle is thicker and difficult to be cut off.

The results also showed a lower harvest success rate (50%) for the modified scenario. The most failure modes were (2) and (6). For instance, the leaves of the lemon tree did not fall when the gripper is open since the leaves were thin and light. The cutting points of the leaves were difficult to be detected due to the random poses. Thus, the leaves may not be removed completely once. In the future work, we will improve the detection of the occluded crops and the leaves, as well as the robot grasping pose.

## 6. Conclusions and Future Work

In this paper, we developed a harvesting robot with a low-cost gripper and proposed a cutting-point detection method for autonomous harvesting. The performance of the proposed harvesting robot was evaluated using a physical robot in different scenarios. We demonstrated the effectiveness of the harvesting robot on different types of crops. System performance results showed that our system can detect the cutting point, cutting the fruit at the appropriate position of the peduncle. Most failures occurred in the attachment stage due to irregular shapes. This problem was avoided by adjusting the directions of crops. In the future, we plan to devise a new approach to detect the cutting pose for irregularly shaped crops.

There are some detachment issues related to the fabrication of the gripper. For instance, the crop did not detach from the peduncle when the gripper is open, while the clamped crop falls due to gravity. Thus, we are working to improve the gripper using metal materials with a better cutting blade, so the gripper would be sufficiently stiff to successfully clamp different types of crops, including one with a thicker peduncle.

In this paper, we mainly focused on the cases where the directions of the crops are downward or tilted. The hypothesis posed in this paper is that a robot capable of reaching targets or leaves without considering obstacles. Human approaches a fruit by pushing a leaf aside or going through the stems without causing any damage to the plant since a damage can seriously hamper plant growth and affect future production of fruit. Inspired by this, we demonstrated how a robot removes leaves to reach the target crop. However, the proposed crop picking process by removing leaves is still in an ideal situation. In the future, we will improve the detection of obstacles, such as unripen crops, leaves and stems. For cases where the direction of a crop parallel to the camera or the peduncle being “occluded” by its flesh, the robot needs to rotate the camera to the next best view [44] to make the cut point of the crop visible. A method to determine the next best view with an optimal grasp [45] based on occlusion information will be considered in the future work.

For the case where stems are viewed as obstacles which are not avoided, we are currently working on developing a vision-guided motion planning method, using a deep reinforcement learning algorithm. This approach allows the robot to reach the desired fruits and avoid obstacles using current visual information. The aforementioned next-best-view-based approach will be combined with the motion planning. Furthermore, we aim to explore the estimation of the locations of crops in three dimensions and predict the occlusion order between objects [46].

As our current robot is inappropriate for use outside, we have not tested our harvesting robot in the field. Our next step is to test more types of crops in the field using a UR5 robot and, in addition, consider the autonomous reconstruction of unknown scenes [47].

## Figures and Tables

**Table 1:** Harvesting results for plastic and real crops.

Success Cases	Plastic Crops							Real Crops			
	Apple (6)	Lemon (3)	Orange (6)	Bitter melon (3)	Grapes (3)	Eggplants (6)	Total	Grapes (7)	Melons (14)	Common figs (10)	Total
**Attachment**	5	2	5	3	2	5	81%	5	11	6	71%
**Detachment**	5	2	4	1	2	5	70%	4	8	5	55%

## Supplementary Materials

The following are available online at https://www.mdpi.com/1424-8220/20/1/93/s1. Video: Autonomous fruit and vegetable harvester with a low-cost gripper using a 3D sensor.
